# Supplementary material for: Accelerated plasma-cell differentiation in Bach2-deficient mouse B cells is caused by altered IRF4 functions
Source: EMBO J. 2024 Apr 11;43(10):1947–64. doi: 10.1038/s44318-024-00077-6 (PMC11099079; doi:10.1038/s44318-024-00077-6)
Supplement: Supplementary file 1 — Appendix [file 44318_2024_77_MOESM1_ESM.pdf]

Appendix

Accelerated plasma cell differentiation in *Bach2*-deficiency is caused by altered IRF4 functions

By Kyoko Ochiai et al.

Appendix Figure S1. 2
Appendix Figure S2. 2
Appendix Figure S3. 3
Appendix Figure S4. 3
Appendix Figure S5. 4
Appendix Figure S6. 4

Appendix Table S1. 5
Appendix Table S2. 6

Cell (Data derived)	Top 1 motif	P-value	% of Targets	% of Background
Ebf1-deficient pre-pro-B cells (Itoh-Nakadai A. Cell Reports 2017)	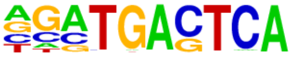	1e-3988	26.88%	2.07%
Activated B1-8^{hi} splenic B cells (This paper)	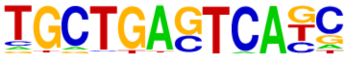	1e-136	55.50%	2.27%

Appendix Figure S1. *De novo* motif analysis of BACH2 ChIP-sequence.
De novo motif were identified using HOMER, and shown with enrichment P-value, % of targets and backgrounds.

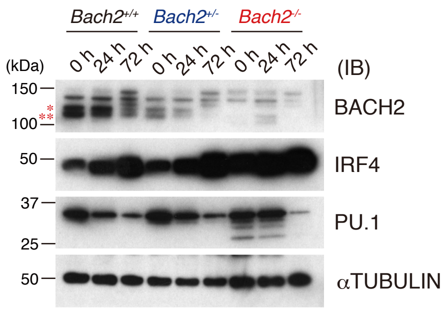

Appendix Figure S2. IRF4 protein was accumulated in *Bach2$^{-/-}$* mice B cells.
Immunoblot analysis of BACH2, IRF4 and PU.1 using B cells purified from *Bach2$^{+/+}$, Bach2$^{+/-}$* and *Bach2$^{-/-}$* mice with B1-8^{hi} background. Cells were stimulated with IL-2, IL-4, IL-5, CD40 ligand and NP40-ficol, and whole cell extracts were prepared at the indicated time. *; p-BACH2, **; BACH2, αTUBULIN; internal control.

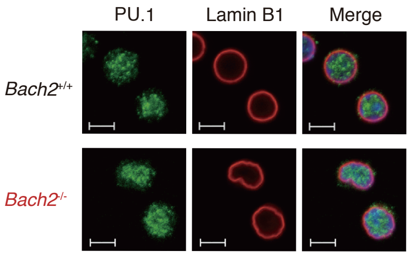

Appendix Figure S3. Nuclear localization of PU.1 in both *Bach2*$^{+/+}$ and *Bach2*$^{-/-}$ mice B cells.
Immunohistochemistry of PU.1 (green) and the nuclear membrane protein LAMIN B1 (red) in B cells purified from *Bach2*$^{+/+}$ and *Bach2*$^{-/-}$ mice with B1-8^{hi} background. Blue indicate nuclei stained with Hoechst 33342. The scales indicate 5 µm.

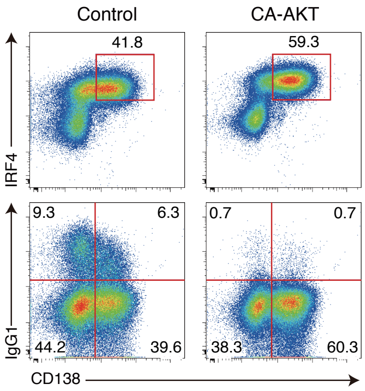

Appendix Figure S4. Tranduction of constitutive active AKT increased the frequency of IRF4^{hi}CD138^{posi} population and inhibited CSR in B1-8^{hi} splenic B cells.
Flow cytometry analysis of B1-8^{hi} splenic B cells transduced with constitutive active AKT (CA-AKT). Data are representative of three independent experiments.

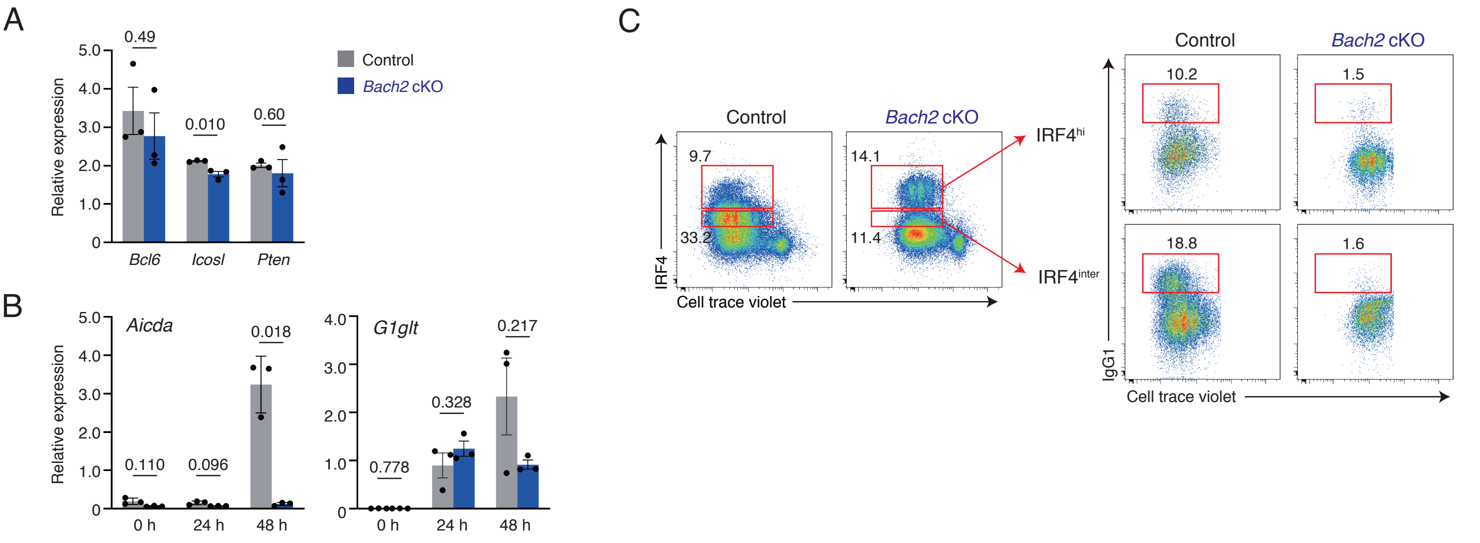

Appendix Figure S5. IRF4 protein was increased upon differentiation of Mb1-Cre:*Bach2*$^{fl/fl}$ mice B cells. Splenic B cells were purified from *Bach2*$^{fl/fl}$ (control) or Mb1-Cre:*Bach2*$^{fl/fl}$ (*Bach2* cKO), followed by stimulating with LPS and IL-4. (**A**) The expression of *Bcl6*, *Icosl* and *Pten* in naive B cells. (**B**) The expression of *Aicda* and germline γ1 transcripts (*G1glt*) at the indicated time. (**C**) IRF4 protein amount with cell division (left), and the frequencies of surface IgG1 and CD138 in IRF4 protein high (IRF4^{hi}) or intermediate (IRF4^{inter}) cells (right) at day 4.

Data information: (A, B) data show the average values ± SD (error bars) acquired from one experiment using three mice for each genotype. P-value by *t*-test using R. (C) Data are representative of each three mice.

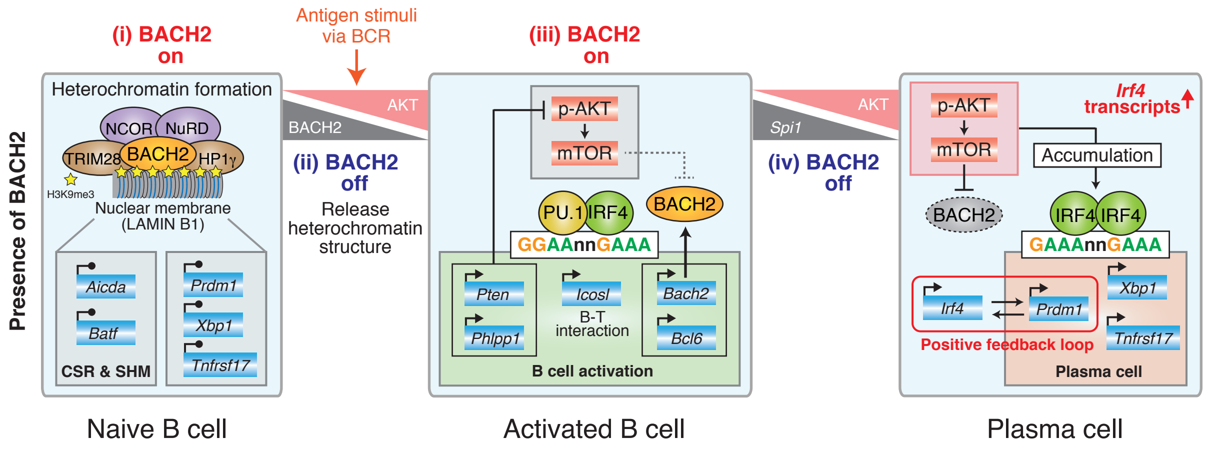

Appendix Figure S6. "BACH2 on-off system" in the promotion of CSR during plasma cell differentiation. BACH2 and IRF4 regulate each other to achieve plasma cell differentiation accomanied with CSR. In naive B cells, BACH2 interacts with H3K9me3 binding proteins, TRIM28 and HP1γ, and the nuclear membrane protein LAMIN B1. BACH2 also interacts with the repressor complexes, NCOR and NuRD, and orchestrates gene repression by heterochromatin formation of target gene loci [(i) **BACH2 on**]. These genes include CSR and SHM related genes, *Aicda* and *Batf*, as well as plasma cell genes, *Prdm1, Xbp1* and *Tnfrsf17 (BCMA)*. BCR stimulation coupled with the AKT signaling inactivates the BACH2 function, resulting in the release of these gene loci from heterochromatin [(ii) **BACH2 off**]. In activated B cells, the PU.1-IRF4 complex induces the expression of *Bach2, Bcl6* and *Icosl.* In this context, they also induce the expression of *Pten* and *Phlpp1,* negative regulators of AKT signaling, and increase the BACH2 function [(iii) **BACH2 on**]. Upon plasma cell differentiation, the transcripts of *Spi1*, encoding PU.1, are reduced, resulting in AKT activation and BACH2 inactivation [(iv) **BACH2 on**]. In plasma cells, the increased AKT activity promotes IRF4 protein accumulation, and the IRF4-PRDM1 positive feedback loop promotes terminal differentiation.

Appendix Table S1. The list of BACH2 complex components detected in GO terms shown in Figure 1D.

Term	GO:0006338 ~chromatin remodeling	GO:0045739 ~positive regulation of DNA repair	GO:0000122 ~negative regulation of transcription from RNA polymerase II promoter	GO:0045944 ~positive regulation of transcription from RNA polymerase II promoter	GO:0006325 ~chromatin organization	GO:0045893 ~positive regulation of transcription, DNA-templated
**Count**	11	8	31	36	16	20
**%**	3.41	2.48	9.60	11.15	4.95	6.19
**PValue**	8.00E-05	1.69E-05	3.69E-04	4.51E-04	2.05E-04	0.0092564
	GeneSymbol	GeneSymbol	GeneSymbol	GeneSymbol	GeneSymbol	GeneSymbol
1	INO80C	INO80C	DDX5	KMT2D	KMT2D	INO80C
2	RBBP4	SMCHD1	SATB1	DHX9	CBX3	SPEN
3	SATB1	PNP	HNRNPU	EIF4A3	SATB1	DDX5
4	RUVBL2	TRIM28	CHD4	HNRNPU	PRKCB	SMAD3
5	RUVBL1	DHX9	ENO1	IKZF3	HNRNPU	NCF1
6	CHD4	RUVBL2	RNF2	IRF2BPL	CHD4	STAT1
7	RBBP7	RUVBL1	BACH2	HELZ2	ARID1A	STAT3
8	ARID1A	RPS3	IRF2BPL	PLAC8	HCFC1	EBF1
9	NPM3		DNAJB1	IKBKB	SMCHD1	NFATC1
10	HCFC1		TRIM28	PTBP1	NCOR1	ARID1A
11	GATAD2A		TPR	RPS6KA3	TRIM28	HCFC1
12			RBBP7	RUVBL2	RBBP4	ILF3
13			SPEN	PCBP1	RUVBL2	ELF1
14			ZHX2	SUMO2	RUVBL1	TRIM28
15			SMAD3	DDX17	RBBP7	RUVBL2
16			CBX3	STAT5A	OGT	RUVBL1
17			STAT1	RBM14		REL
18			NFATC1	PPP1R12A		IRF8
19			ARID1A	SMAD3		CRLF3
20			YWHAZ	STAT1		SNX5
21			HCFC1	STAT3		
22			GATAD2A	EBF1		
23			NCOR1	NFATC1		
24			HNRNPK	SAFB		
25			CNOT1	NFKB2		
26			CNOT2	HCFC1		
27			SARNP	ELF1		
28			HNRNPA2B1	RBMXL1		
29			IRF8	HNRNPK		
30			TAGLN3	PSMC3		
31			EZR	AGO2		
32				REL		
33				IRF8		
34				CRLF3		
35				PTMA		
36				OGT		

Appendix Table S2. Primers used in this study.

RT-PCR

	Forward	Reverse
Bach2	CGCTGTCGAAAGAGGAAGCTGGAC	CCTGGATCTGCTCTGGACTCTGGA
Prdm1	CCCTCTGAAGAAACAGAATG	GCTTGTGCTGCTAAATCTCT
Irf4	TGTGCTCTGAACAAGAGCAAT	TATGAACCTGCTGGGCTGG
Batf	CATCTGATGATGTGAGGAAA	GAGCTGTTTGATCTCTTTGC
Aicda	AGGGAGTCAAGAAAGTCACG	CAGGAGGTGGCACTATCTCT
G1glt	GGCCCTTCCAGATCTTTGAG	GGATCCAGAGTTCCAGGTCACT
Bcl6	GCAGTTTAGAGCCCATAAGA	GTACATGAAGTCCAGGAGGA
Icosl	TCTGGTCTTGGTCTGTTCTT	GGAGATTGTAAGGCAGGTA
Pten	GAGATCGTTAGCAGAAACAAA	CAGGAAATCCCATAGCAATA
Phlpp1	CTTTCACGGAGTACTTACGG	ATGTTCAAGGCTACAACAGG
Xbp1	AAAACAGAGTAGCAGCGCAG	TTTCTAGCTGGAGTTTGTGG
Hmox1	GGGTGACAGAAGAGGCTAAG	GTGTCTGGGATGAGCTAGTG
β-2microglobulin (β2m)	AGACTGATACATACGCCTGCA	GCAGGTTCAAATGAATCTTCAG

ChIP-PCR

	Forward	Reverse
Bcl6	TTTGATGTCACCACTCACC	GTTTGACATCGACACCAGA
Icosl	GTTCAGACCTCTTAAGAC	GAGTGCAGTAGATGTTGAG
Pten	GGATATAATCAGCCCGAGA	CTCTGAGACCTAGCACAGGA
Phlpp1	GATATTGGATCCTCTGGAACT	GTACCACTGGGTACTCTTCAA
k3E	TCATAGCTACCGTCACACTG	AACAGATGTGCCTAAGGTTC
Prdm1 cns9	AAGCTGCTAAGTGGGAGAGT	GCATAATTTAGCGTTTGGTG
Prdm1 cns1	CCCTGACAATGTTTGTCCTA	CGGACAAGAAGAGCAAGTTA
