# Supplementary figures and images for: Accelerated plasma-cell differentiation in Bach2-deficient mouse B cells is caused by altered IRF4 functions
Source: EMBO J. 2024 Apr 11;43(10):1947–64. doi: 10.1038/s44318-024-00077-6 (PMC11099079; doi:10.1038/s44318-024-00077-6)

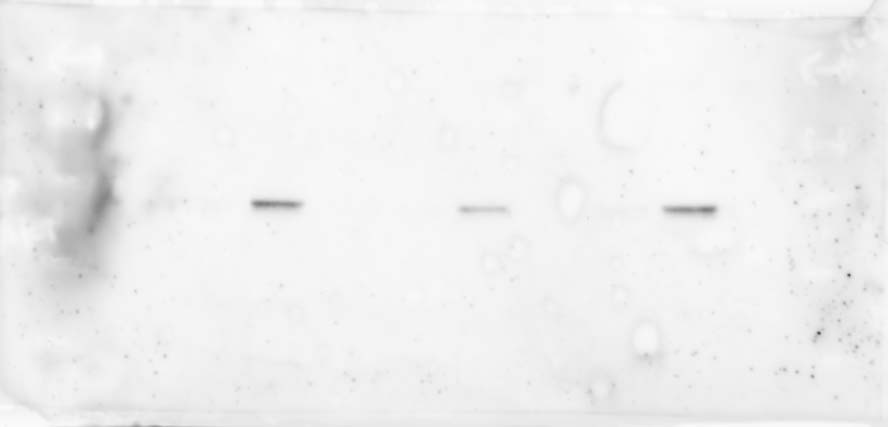

Supplement: Supplementary file 6 — Source data Fig. 1 [file 44318_2024_77_MOESM6_ESM.zip › Figure 1/1F/p-AKT.tif]

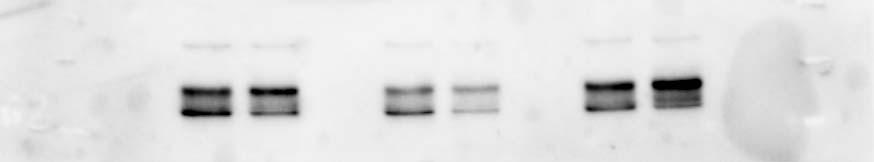

Supplement: Supplementary file 6 — Source data Fig. 1 [file 44318_2024_77_MOESM6_ESM.zip › Figure 1/1F/Bach2.tif]

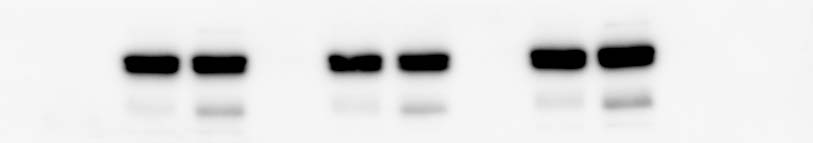

Supplement: Supplementary file 6 — Source data Fig. 1 [file 44318_2024_77_MOESM6_ESM.zip › Figure 1/1F/Trim28.tif]

One experiment using independent three mice.

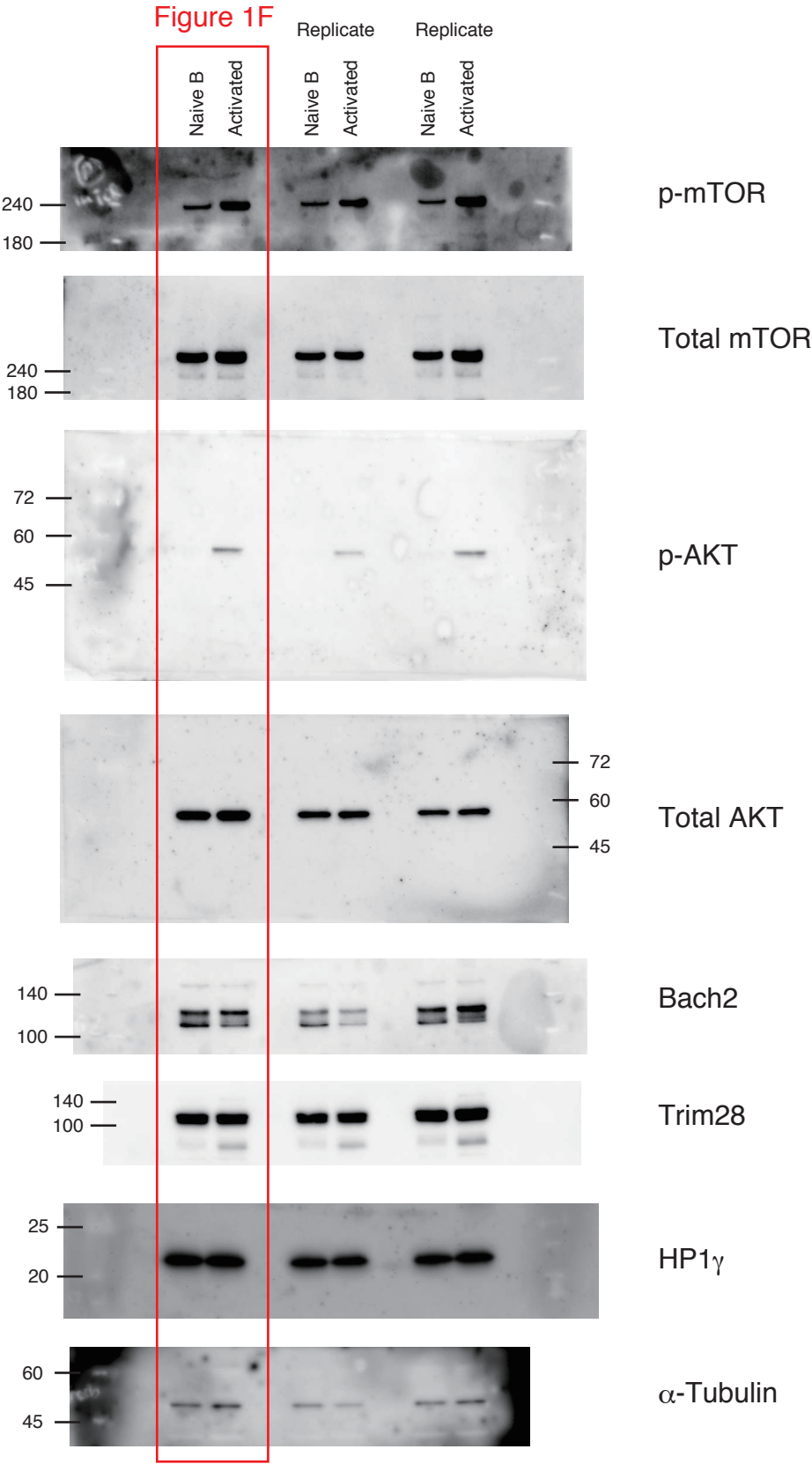

Supplement: Supplementary file 6 — Source data Fig. 1 [file 44318_2024_77_MOESM6_ESM.zip › Figure 1/1F/README_1F.pdf]

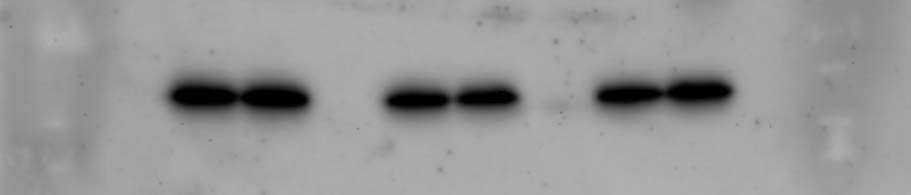

Supplement: Supplementary file 6 — Source data Fig. 1 [file 44318_2024_77_MOESM6_ESM.zip › Figure 1/1F/HP1g.tif]

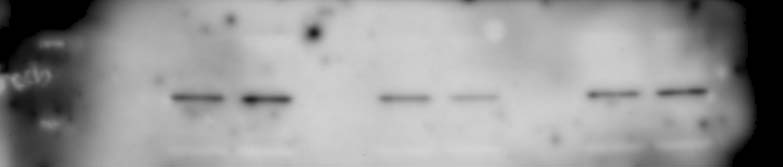

Supplement: Supplementary file 6 — Source data Fig. 1 [file 44318_2024_77_MOESM6_ESM.zip › Figure 1/1F/a-tubulin.tif]

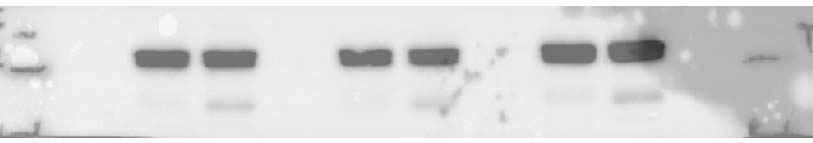

Supplement: Supplementary file 6 — Source data Fig. 1 [file 44318_2024_77_MOESM6_ESM.zip › Figure 1/1F/Trim28withMarker.tif]

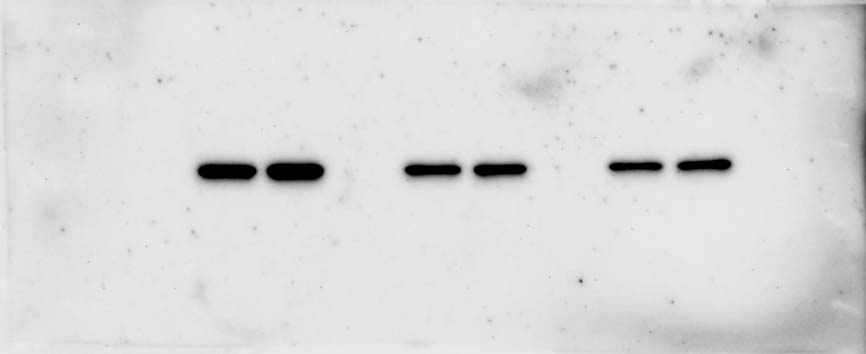

Supplement: Supplementary file 6 — Source data Fig. 1 [file 44318_2024_77_MOESM6_ESM.zip › Figure 1/1F/Total_AKT.tif]

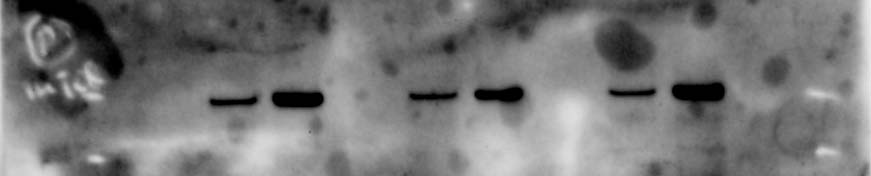

Supplement: Supplementary file 6 — Source data Fig. 1 [file 44318_2024_77_MOESM6_ESM.zip › Figure 1/1F/p-mTOR.tif]

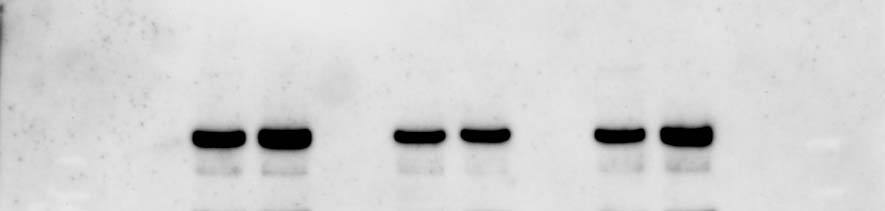

Supplement: Supplementary file 6 — Source data Fig. 1 [file 44318_2024_77_MOESM6_ESM.zip › Figure 1/1F/Total_mTOR.tif]

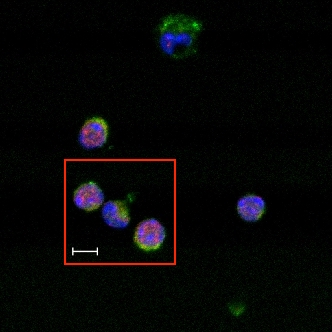

Supplement: Supplementary file 6 — Source data Fig. 1 [file 44318_2024_77_MOESM6_ESM.zip › Figure 1/1H/ActivatedB/2_Merge.jpg]

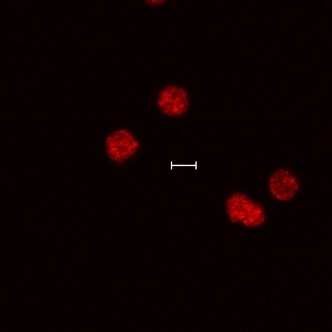

Supplement: Supplementary file 6 — Source data Fig. 1 [file 44318_2024_77_MOESM6_ESM.zip › Figure 1/1H/ActivatedB/1_HP1g.jpg]

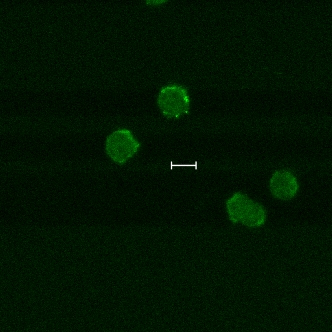

Supplement: Supplementary file 6 — Source data Fig. 1 [file 44318_2024_77_MOESM6_ESM.zip › Figure 1/1H/ActivatedB/1_Bach2.jpg]

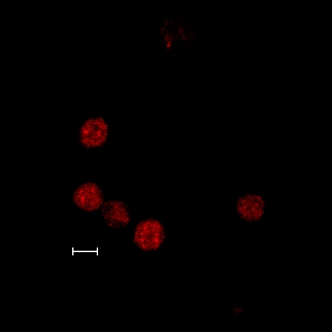

Supplement: Supplementary file 6 — Source data Fig. 1 [file 44318_2024_77_MOESM6_ESM.zip › Figure 1/1H/ActivatedB/2_HP1g.jpg]

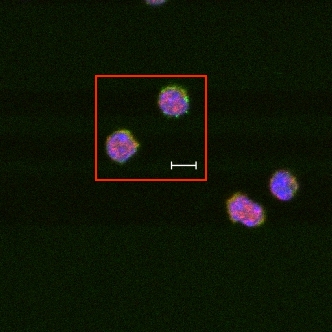

Supplement: Supplementary file 6 — Source data Fig. 1 [file 44318_2024_77_MOESM6_ESM.zip › Figure 1/1H/ActivatedB/1_Merge.jpg]

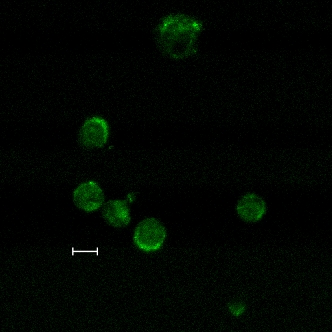

Supplement: Supplementary file 6 — Source data Fig. 1 [file 44318_2024_77_MOESM6_ESM.zip › Figure 1/1H/ActivatedB/2_Bach2.jpg]

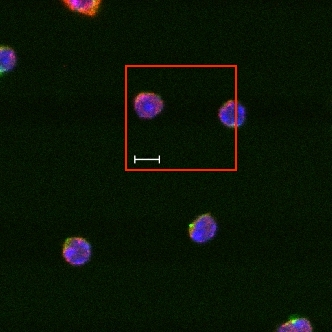

Supplement: Supplementary file 6 — Source data Fig. 1 [file 44318_2024_77_MOESM6_ESM.zip › Figure 1/1H/NaiveB/2_Merge.jpg]

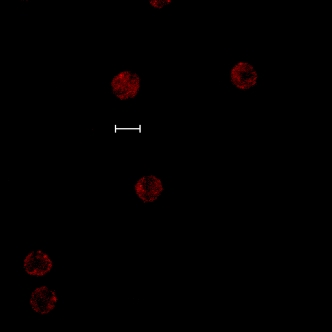

Supplement: Supplementary file 6 — Source data Fig. 1 [file 44318_2024_77_MOESM6_ESM.zip › Figure 1/1H/NaiveB/1_HP1g.jpg]

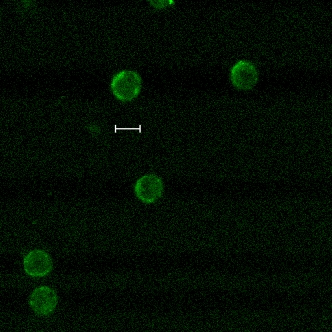

Supplement: Supplementary file 6 — Source data Fig. 1 [file 44318_2024_77_MOESM6_ESM.zip › Figure 1/1H/NaiveB/1_Bach2.jpg]

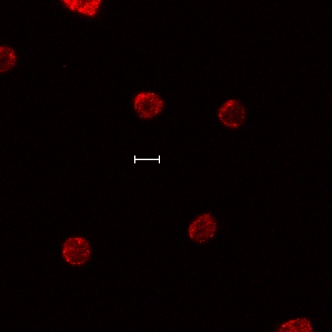

Supplement: Supplementary file 6 — Source data Fig. 1 [file 44318_2024_77_MOESM6_ESM.zip › Figure 1/1H/NaiveB/2_HP1g.jpg]

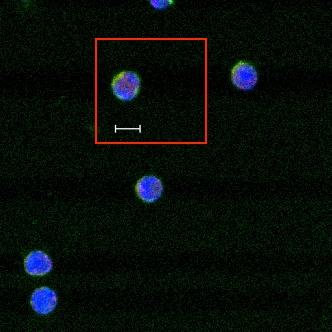

Supplement: Supplementary file 6 — Source data Fig. 1 [file 44318_2024_77_MOESM6_ESM.zip › Figure 1/1H/NaiveB/1_Merge.jpg]

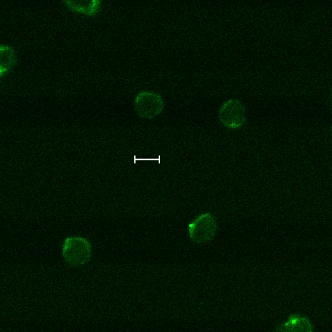

Supplement: Supplementary file 6 — Source data Fig. 1 [file 44318_2024_77_MOESM6_ESM.zip › Figure 1/1H/NaiveB/2_Bach2.jpg]

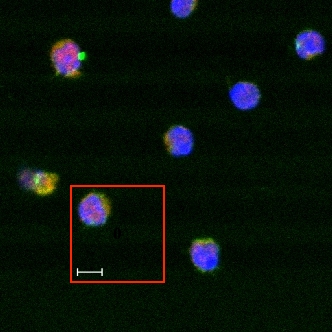

Supplement: Supplementary file 6 — Source data Fig. 1 [file 44318_2024_77_MOESM6_ESM.zip › Figure 1/1G/ActivatedB/2_Merge.jpg]

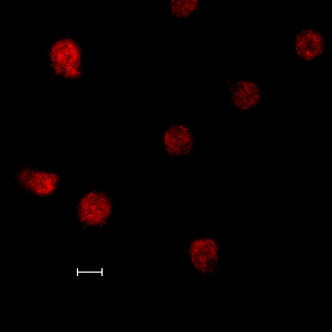

Supplement: Supplementary file 6 — Source data Fig. 1 [file 44318_2024_77_MOESM6_ESM.zip › Figure 1/1G/ActivatedB/2_Trim28.jpg]

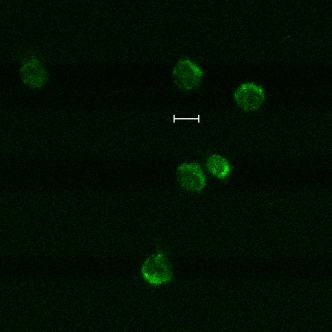

Supplement: Supplementary file 6 — Source data Fig. 1 [file 44318_2024_77_MOESM6_ESM.zip › Figure 1/1G/ActivatedB/1_Bach2.jpg]

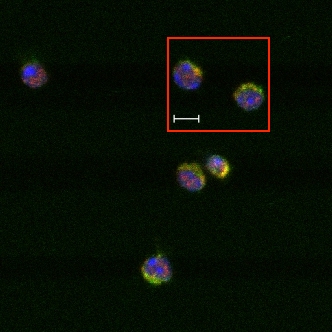

Supplement: Supplementary file 6 — Source data Fig. 1 [file 44318_2024_77_MOESM6_ESM.zip › Figure 1/1G/ActivatedB/1_Merge.jpg]

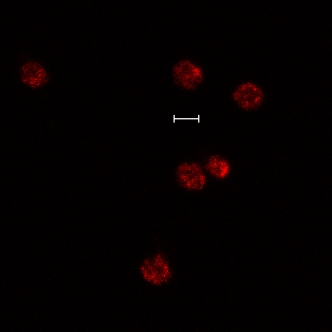

Supplement: Supplementary file 6 — Source data Fig. 1 [file 44318_2024_77_MOESM6_ESM.zip › Figure 1/1G/ActivatedB/1_Trim28.jpg]

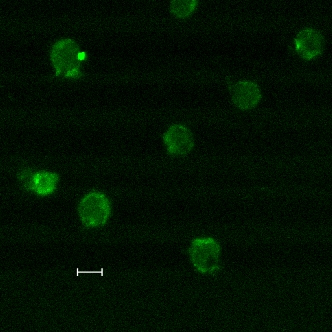

Supplement: Supplementary file 6 — Source data Fig. 1 [file 44318_2024_77_MOESM6_ESM.zip › Figure 1/1G/ActivatedB/2_Bach2.jpg]

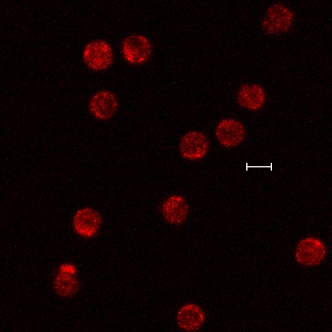

Supplement: Supplementary file 6 — Source data Fig. 1 [file 44318_2024_77_MOESM6_ESM.zip › Figure 1/1G/NaiveB/Trim28.jpg]

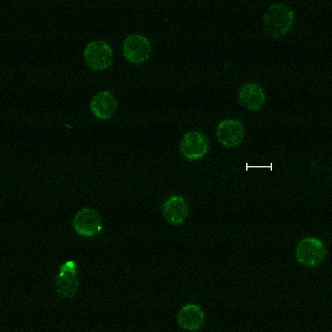

Supplement: Supplementary file 6 — Source data Fig. 1 [file 44318_2024_77_MOESM6_ESM.zip › Figure 1/1G/NaiveB/Bach2.jpg]

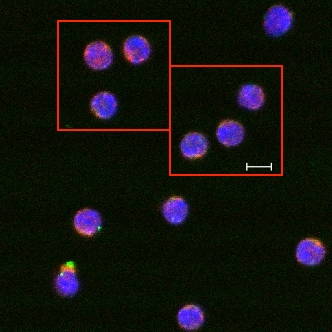

Supplement: Supplementary file 6 — Source data Fig. 1 [file 44318_2024_77_MOESM6_ESM.zip › Figure 1/1G/NaiveB/Merge.jpg]

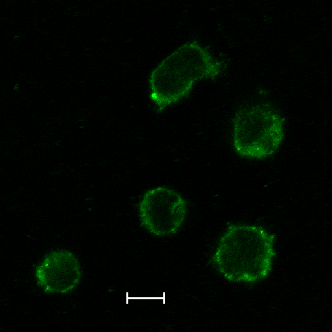

Supplement: Supplementary file 6 — Source data Fig. 1 [file 44318_2024_77_MOESM6_ESM.zip › Figure 1/1B/ActivatedB/1_BACH2.tif]

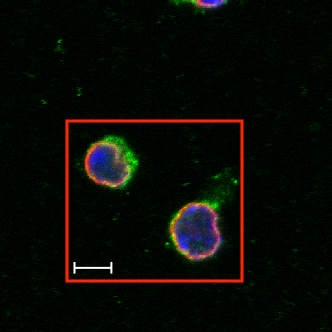

Supplement: Supplementary file 6 — Source data Fig. 1 [file 44318_2024_77_MOESM6_ESM.zip › Figure 1/1B/ActivatedB/2_Merge.tif]

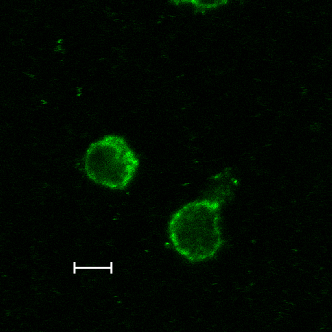

Supplement: Supplementary file 6 — Source data Fig. 1 [file 44318_2024_77_MOESM6_ESM.zip › Figure 1/1B/ActivatedB/2_BACH2.tif]

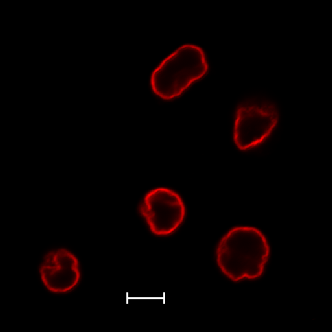

Supplement: Supplementary file 6 — Source data Fig. 1 [file 44318_2024_77_MOESM6_ESM.zip › Figure 1/1B/ActivatedB/1_LaminB1.tif]

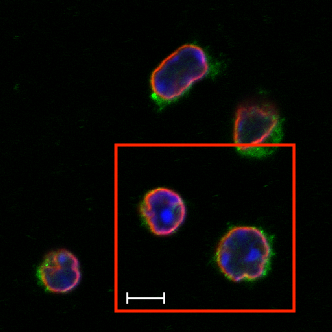

Supplement: Supplementary file 6 — Source data Fig. 1 [file 44318_2024_77_MOESM6_ESM.zip › Figure 1/1B/ActivatedB/1_Merge.tif]

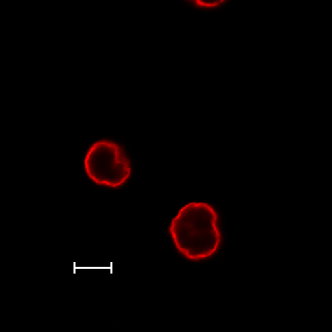

Supplement: Supplementary file 6 — Source data Fig. 1 [file 44318_2024_77_MOESM6_ESM.zip › Figure 1/1B/ActivatedB/2_LaminB1.tif]

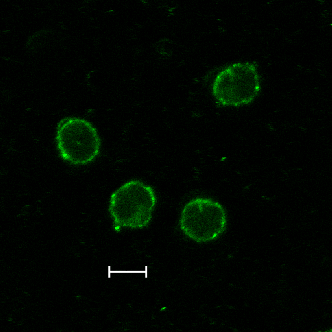

Supplement: Supplementary file 6 — Source data Fig. 1 [file 44318_2024_77_MOESM6_ESM.zip › Figure 1/1B/NaiveB/1_BACH2.tif]

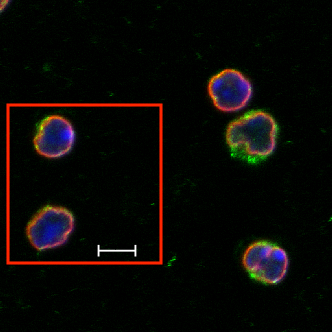

Supplement: Supplementary file 6 — Source data Fig. 1 [file 44318_2024_77_MOESM6_ESM.zip › Figure 1/1B/NaiveB/2_Merge.tif]

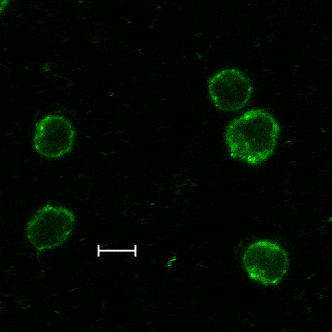

Supplement: Supplementary file 6 — Source data Fig. 1 [file 44318_2024_77_MOESM6_ESM.zip › Figure 1/1B/NaiveB/2_BACH2.tif]

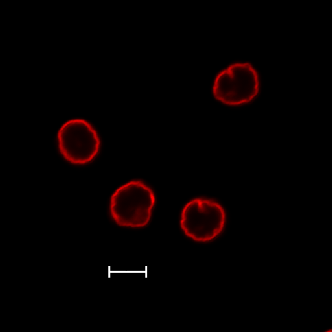

Supplement: Supplementary file 6 — Source data Fig. 1 [file 44318_2024_77_MOESM6_ESM.zip › Figure 1/1B/NaiveB/1_LaminB1.tif]

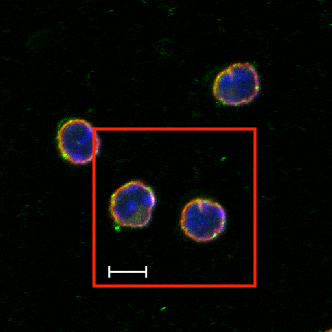

Supplement: Supplementary file 6 — Source data Fig. 1 [file 44318_2024_77_MOESM6_ESM.zip › Figure 1/1B/NaiveB/1_Merge.tif]

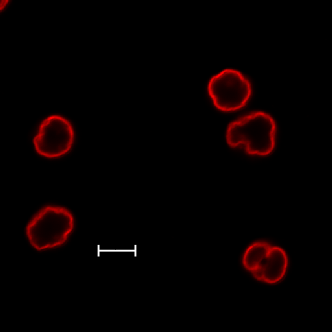

Supplement: Supplementary file 6 — Source data Fig. 1 [file 44318_2024_77_MOESM6_ESM.zip › Figure 1/1B/NaiveB/2_LaminB1.tif]

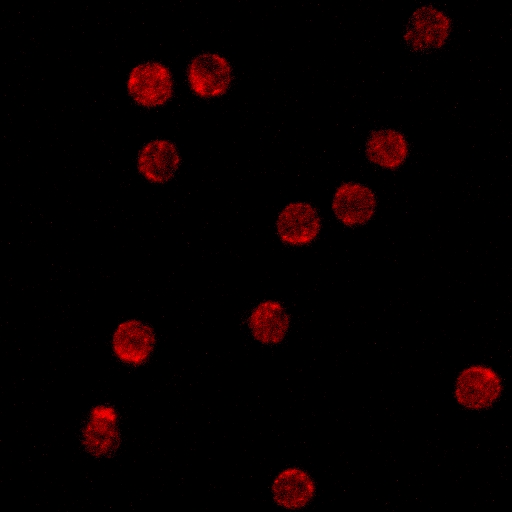

Supplement: Supplementary file 6 — Source data Fig. 1 [file 44318_2024_77_MOESM6_ESM.zip › Figure 1/1G/NaiveB/replicate/Trim28.jpg]

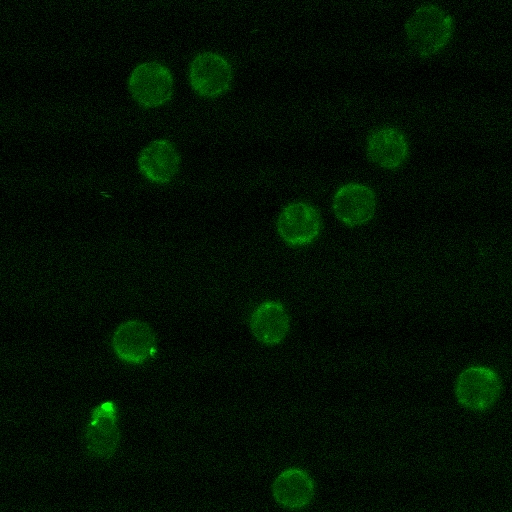

Supplement: Supplementary file 6 — Source data Fig. 1 [file 44318_2024_77_MOESM6_ESM.zip › Figure 1/1G/NaiveB/replicate/Bach2.jpg]

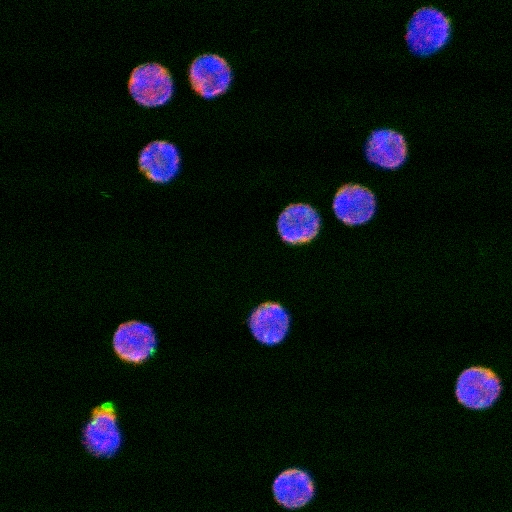

Supplement: Supplementary file 6 — Source data Fig. 1 [file 44318_2024_77_MOESM6_ESM.zip › Figure 1/1G/NaiveB/replicate/Merge.jpg]

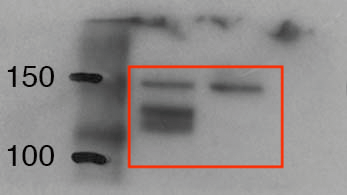

Supplement: Supplementary file 7 — Source data Fig. 3 [file 44318_2024_77_MOESM7_ESM.zip › Figure 3/3C/Bach2.tif]

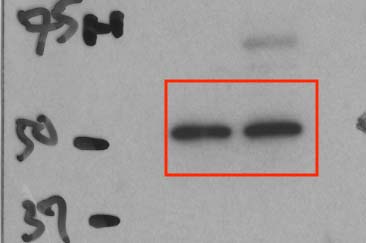

Supplement: Supplementary file 7 — Source data Fig. 3 [file 44318_2024_77_MOESM7_ESM.zip › Figure 3/3C/aTubulin.jpeg]

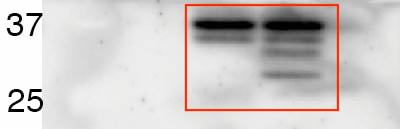

Supplement: Supplementary file 7 — Source data Fig. 3 [file 44318_2024_77_MOESM7_ESM.zip › Figure 3/3C/PU1.tif]

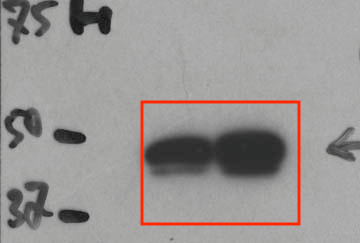

Supplement: Supplementary file 7 — Source data Fig. 3 [file 44318_2024_77_MOESM7_ESM.zip › Figure 3/3C/IRF4.tif]

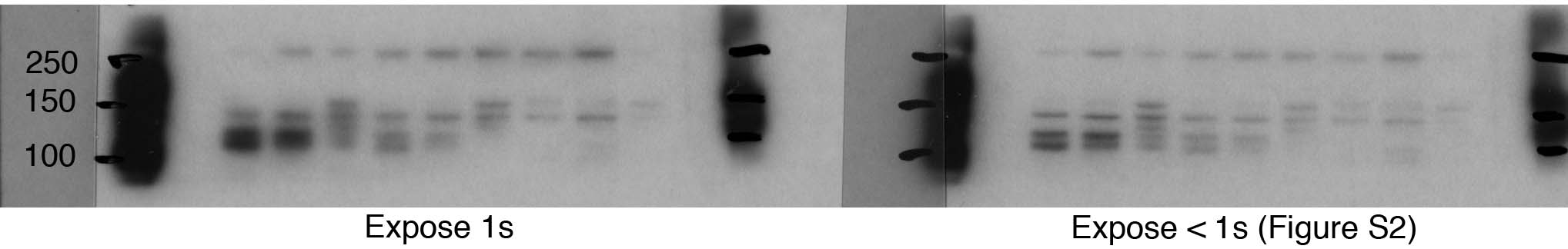

Supplement: Supplementary file 7 — Source data Fig. 3 [file 44318_2024_77_MOESM7_ESM.zip › Figure 3/3C/replicate_asFigS2/Bach2.tif]

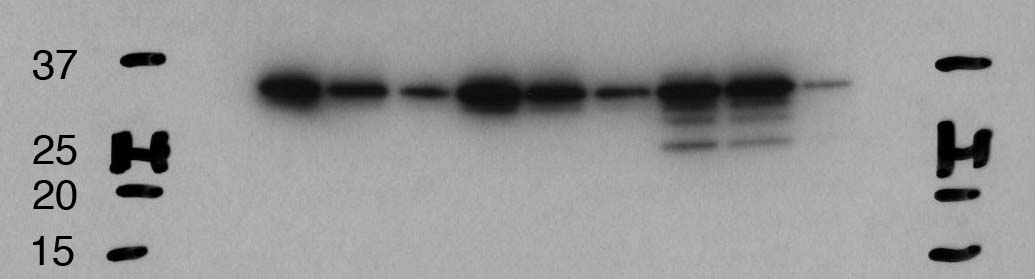

Supplement: Supplementary file 7 — Source data Fig. 3 [file 44318_2024_77_MOESM7_ESM.zip › Figure 3/3C/replicate_asFigS2/PU1.tif]

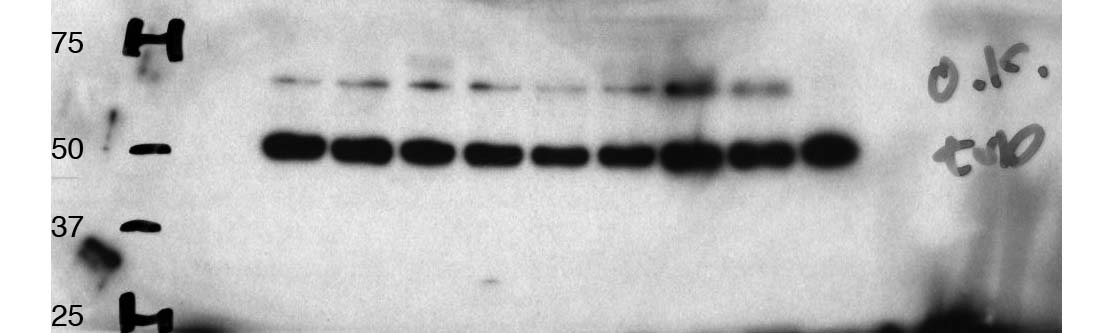

Supplement: Supplementary file 7 — Source data Fig. 3 [file 44318_2024_77_MOESM7_ESM.zip › Figure 3/3C/replicate_asFigS2/aTubulin.tif]

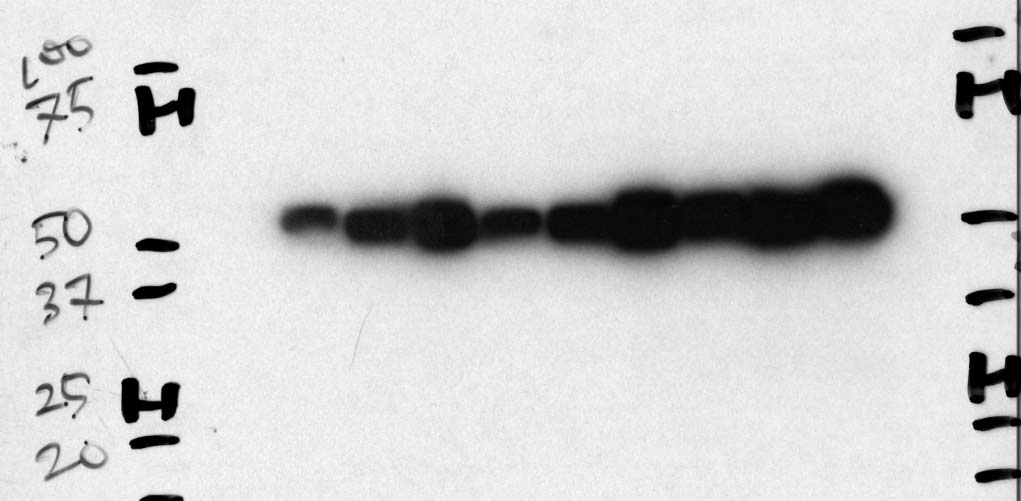

Supplement: Supplementary file 7 — Source data Fig. 3 [file 44318_2024_77_MOESM7_ESM.zip › Figure 3/3C/replicate_asFigS2/IRF4.tif]

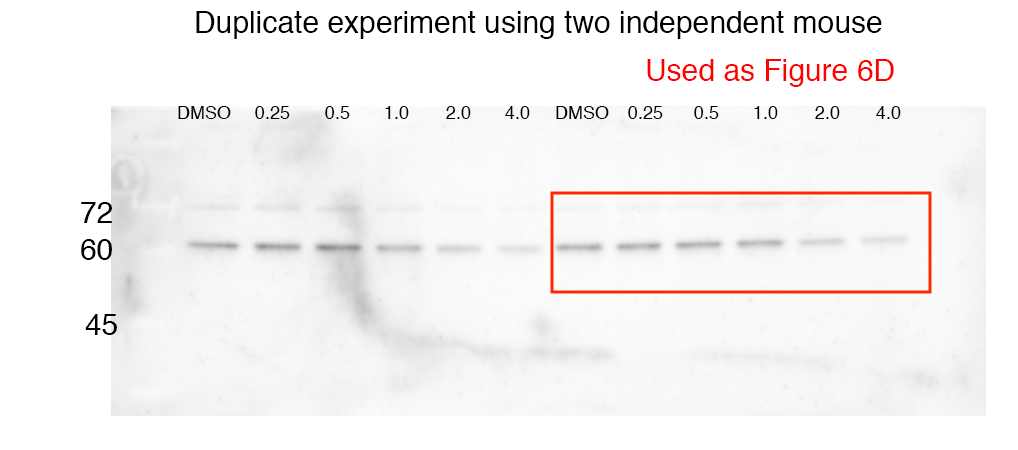

Supplement: Supplementary file 10 — Source data Fig. 6 [file 44318_2024_77_MOESM10_ESM.zip › Figure 6/6D/p-AKT.tif]

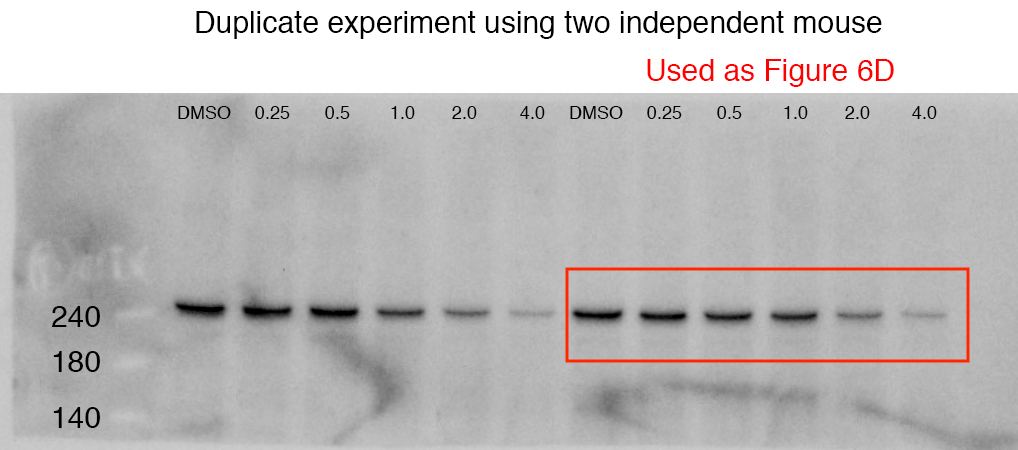

Supplement: Supplementary file 10 — Source data Fig. 6 [file 44318_2024_77_MOESM10_ESM.zip › Figure 6/6D/p-mTOR.tif]

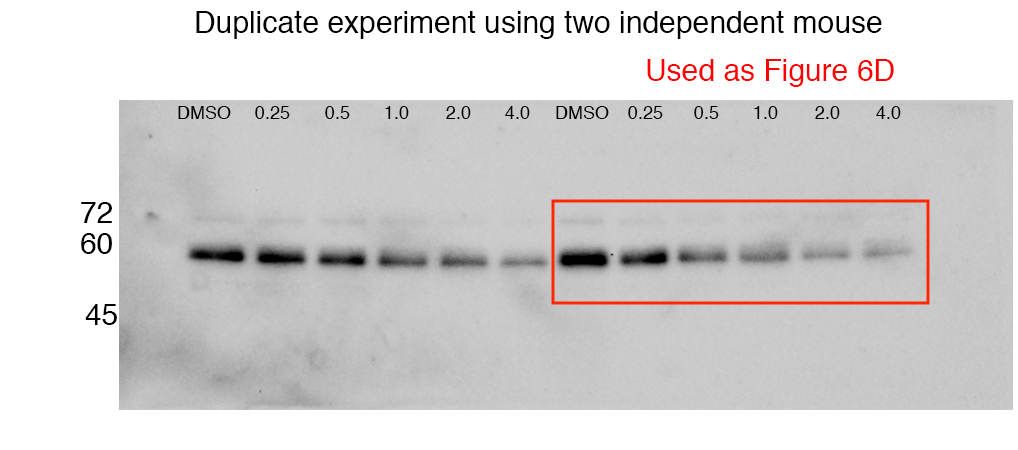

Supplement: Supplementary file 10 — Source data Fig. 6 [file 44318_2024_77_MOESM10_ESM.zip › Figure 6/6D/total_AKT.tif]

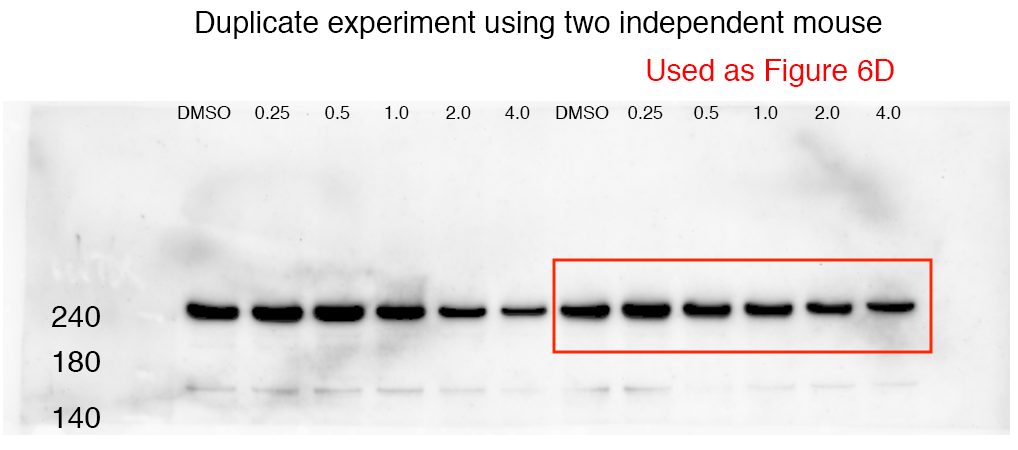

Supplement: Supplementary file 10 — Source data Fig. 6 [file 44318_2024_77_MOESM10_ESM.zip › Figure 6/6D/total_mTOR.tif]

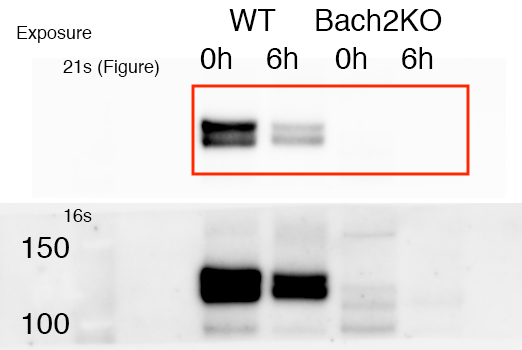

Supplement: Supplementary file 10 — Source data Fig. 6 [file 44318_2024_77_MOESM10_ESM.zip › Figure 6/6F/Bach2.tif]

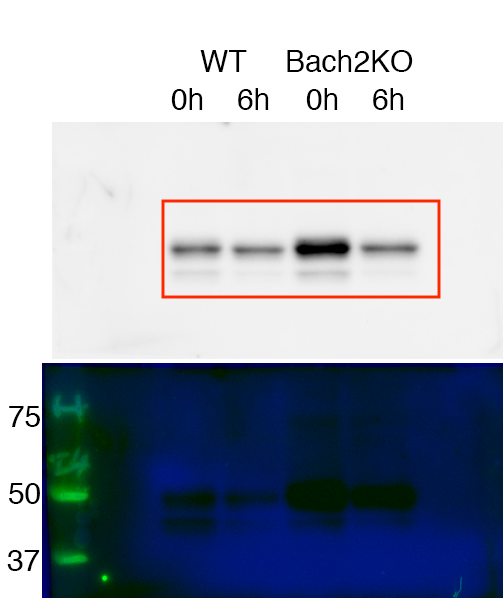

Supplement: Supplementary file 10 — Source data Fig. 6 [file 44318_2024_77_MOESM10_ESM.zip › Figure 6/6F/IRF4.tif]

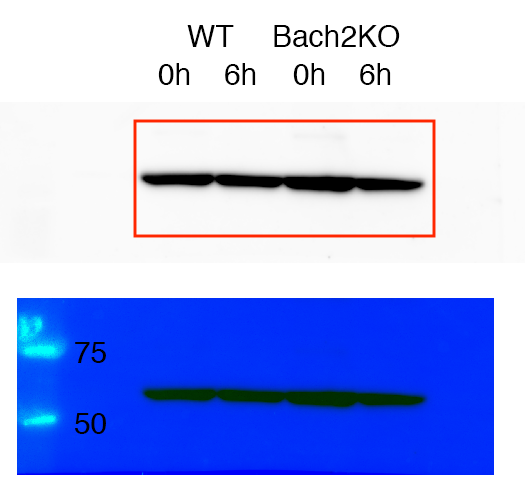

Supplement: Supplementary file 10 — Source data Fig. 6 [file 44318_2024_77_MOESM10_ESM.zip › Figure 6/6F/b-ACTIN.tif]

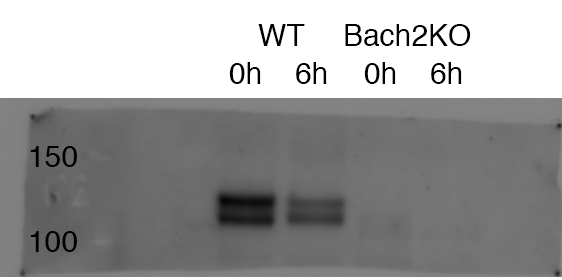

Supplement: Supplementary file 10 — Source data Fig. 6 [file 44318_2024_77_MOESM10_ESM.zip › Figure 6/6F/replicate/Bach2.tif]

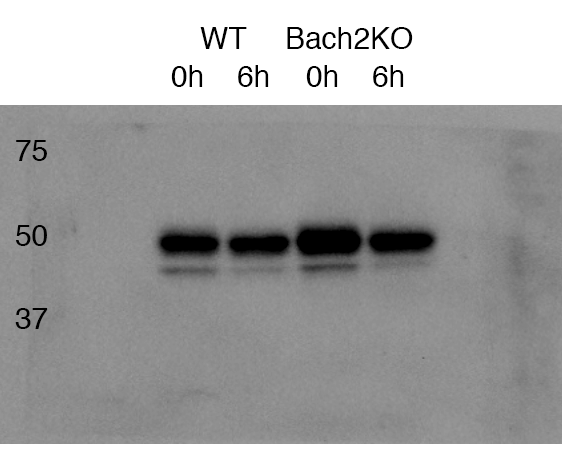

Supplement: Supplementary file 10 — Source data Fig. 6 [file 44318_2024_77_MOESM10_ESM.zip › Figure 6/6F/replicate/IRF4.tif]

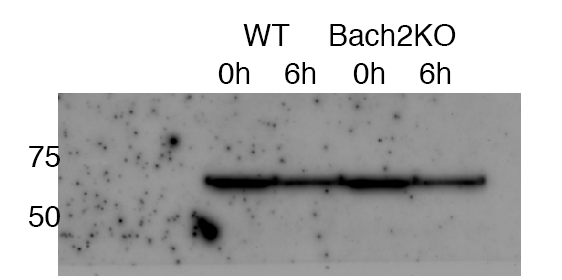

Supplement: Supplementary file 10 — Source data Fig. 6 [file 44318_2024_77_MOESM10_ESM.zip › Figure 6/6F/replicate/b-ACTIN.tif]

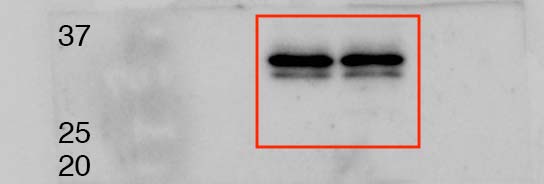

Supplement: Supplementary file 11 — Source data Fig. 7 [file 44318_2024_77_MOESM11_ESM.zip › Figure 7/7C/PU1.jpeg]

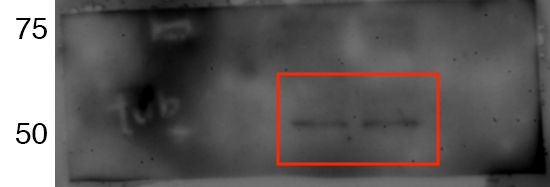

Supplement: Supplementary file 11 — Source data Fig. 7 [file 44318_2024_77_MOESM11_ESM.zip › Figure 7/7C/aTubulin.tif]

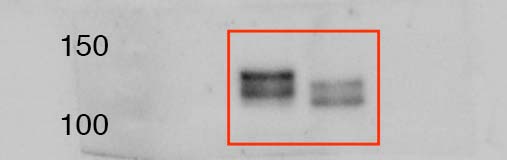

Supplement: Supplementary file 11 — Source data Fig. 7 [file 44318_2024_77_MOESM11_ESM.zip › Figure 7/7C/Bach2.jpeg]

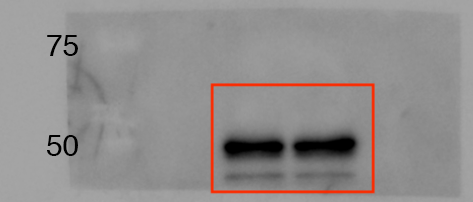

Supplement: Supplementary file 11 — Source data Fig. 7 [file 44318_2024_77_MOESM11_ESM.zip › Figure 7/7C/IRF4.tif]

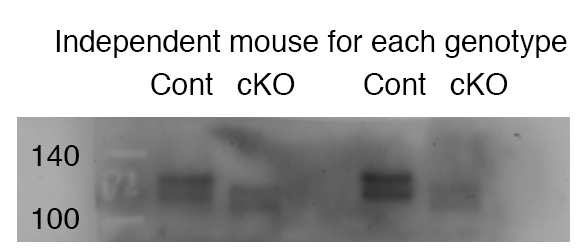

Supplement: Supplementary file 11 — Source data Fig. 7 [file 44318_2024_77_MOESM11_ESM.zip › Figure 7/7C/replicates/Bach2.tif]

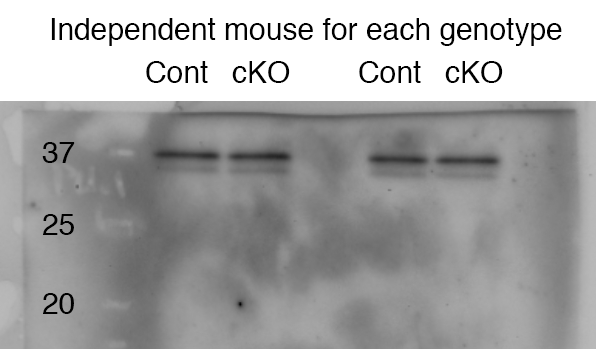

Supplement: Supplementary file 11 — Source data Fig. 7 [file 44318_2024_77_MOESM11_ESM.zip › Figure 7/7C/replicates/PU1.tif]

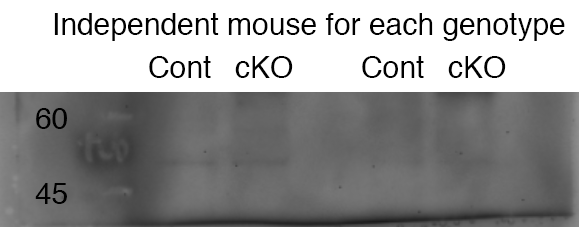

Supplement: Supplementary file 11 — Source data Fig. 7 [file 44318_2024_77_MOESM11_ESM.zip › Figure 7/7C/replicates/aTubulin.tif]

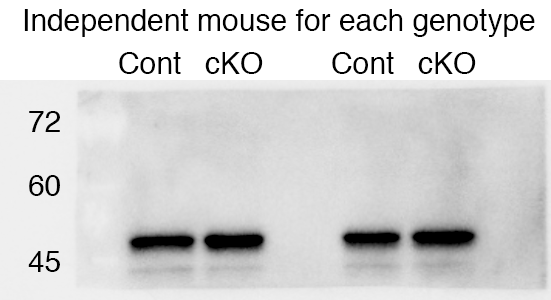

Supplement: Supplementary file 11 — Source data Fig. 7 [file 44318_2024_77_MOESM11_ESM.zip › Figure 7/7C/replicates/IRF4.tif]
