# Supplementary material for: Accelerated plasma-cell differentiation in Bach2-deficient mouse B cells is caused by altered IRF4 functions
Source: EMBO J. 2024 Apr 11;43(10):1947–64. doi: 10.1038/s44318-024-00077-6 (PMC11099079; doi:10.1038/s44318-024-00077-6)
Supplement: Supplementary file 8 — Source data Fig. 5J,L [file 44318_2024_77_MOESM8_ESM.zip › Figure 5J,L/5J/README_5J.rtf]

Staining information

surface B220-APC
surface CD19-PerCP
intracellular pAKT-Alexa488

Data was sorted as following.
	Remove duplicate cells
	Sort B220+CD19+ cells
	Shown pAKT by histogram
