# Supplementary material for: Accelerated plasma-cell differentiation in Bach2-deficient mouse B cells is caused by altered IRF4 functions
Source: EMBO J. 2024 Apr 11;43(10):1947–64. doi: 10.1038/s44318-024-00077-6 (PMC11099079; doi:10.1038/s44318-024-00077-6)
Supplement: Supplementary file 9 — Source data Fig. 5M,N,O [file 44318_2024_77_MOESM9_ESM.zip › Figure 5M,N,O/5M/README_5M.rtf]

Staining information

Transduced cells (dsRed)
intracellular pAKT-Fitc
