# Supplementary material for: Accelerated plasma-cell differentiation in Bach2-deficient mouse B cells is caused by altered IRF4 functions
Source: EMBO J. 2024 Apr 11;43(10):1947–64. doi: 10.1038/s44318-024-00077-6 (PMC11099079; doi:10.1038/s44318-024-00077-6)
Supplement: Supplementary file 9 — Source data Fig. 5M,N,O [file 44318_2024_77_MOESM9_ESM.zip › Figure 5M,N,O/5O/README_5O.rtf]

Staining information

Transduced cells (GFP)
surface CD138
surface IgG1-PerCP
intracellular IRF4

Data was sorted as following.
	Remove duplicate cells
	Sort GFP+ cells
	Shown by CD138+ (X-axis) and IRF4 (Y-axis)
