# Supplementary material for: Accelerated plasma-cell differentiation in Bach2-deficient mouse B cells is caused by altered IRF4 functions
Source: EMBO J. 2024 Apr 11;43(10):1947–64. doi: 10.1038/s44318-024-00077-6 (PMC11099079; doi:10.1038/s44318-024-00077-6)
Supplement: Supplementary file 11 — Source data Fig. 7 [file 44318_2024_77_MOESM11_ESM.zip › Figure 7/7D/README_7D.rtf]

Staining informationsurface B220-APCintracellular pAKT-Alexa488
