# Supplementary material for: Accelerated plasma-cell differentiation in Bach2-deficient mouse B cells is caused by altered IRF4 functions
Source: EMBO J. 2024 Apr 11;43(10):1947–64. doi: 10.1038/s44318-024-00077-6 (PMC11099079; doi:10.1038/s44318-024-00077-6)
Supplement: Supplementary file 11 — Source data Fig. 7 [file 44318_2024_77_MOESM11_ESM.zip › Figure 7/7A/README_7A.rtf]

Staining information

Dapi
surface B220-APC
surface CD19-PerCP
CD21-Fitc
CD23-PE

Data was sorted as following.
	Remove duplicate cells
	Remove dead cells (sort Dapi-)
	Sort B220+CD19+ cells
	Shown by CD21 (X-axis) and CD23 (Y-axis)

Experimental lot 1; WT, KO1, KO2, KO3, wKO4
Experimental lot 2; WT1,WT2,WT3
